# Agreement and repeatability of intraocular pressure measurements between SK-5500B and Icare IC200 tonometers in sitting and supine positions: a comparative study

**DOI:** 10.3389/fphys.2025.1746123

**Published:** 2026-01-08

**Authors:** Nan Li, Yucong Chen, Kunliang Qiu, Zhiqiang Guan

**Affiliations:** 1 Joint Shantou International Eye Center of Shantou University and The Chinese University of Hong Kong, Shantou, China; 2 Shantou University Medical College, Shantou, China

**Keywords:** agreement, Icare ic200, intraocular pressure, rebound tonometry, repeatability, SK-5500B

## Abstract

**Introduction:**

Accurate intraocular pressure (IOP) measurement is vital in glaucoma management, yet the gold standard (Goldmann applanation tonometry) is limited to seated measurements. Rebound tonometers like the Icare IC200 and the newer SK-5500B offer portability for assessing posture-induced IOP changes, but their measurement consistency requires validation. This study aims to evaluate the agreement and repeatability of IOP measurements between the SK-5500B and Icare IC200 tonometers in sitting and supine positions.

**Methods:**

This prospective study recruited 59 subjects (97 eyes). IOP was measured in both sitting and supine positions by two masked operators using the SK-5500B and Icare IC200 tonometers in a randomized sequence. Subjects were categorized into four groups based on their baseline non-contact tonometry (NCT) readings (<10 mmHg, 10–21 mmHg, 22–30 mmHg, and >30 mmHg). Agreement was assessed using Pearson’s correlation and Bland-Altman analysis. Correlation was evaluated using Pearson’s correlation coefficient (r). Intra- and inter-operator repeatability were determined by within-subject standard deviation (Sw), coefficient of variation (CV), and intraclass correlation coefficient (ICC). The correlation between the device and central corneal thickness (CCT) or axial length (AL) was also analyzed.

**Results:**

The SK-5500B and Icare IC200 showed a strong positive correlation across all conditions (both positions and both operators) (r ≥ 0.90, *P* < 0.0001). Bland-Altman analysis demonstrated small mean bias, but the limits of agreement were relatively wide between the two tonometers. Both tonometers exhibited excellent intra-operator (ICC ≥ 0.99) and inter-operator (ICC ≥ 0.99) repeatability. IOP measurements from both tonometers did not show a significant correlation with CCT or AL (*P* > 0.05).

**Conclusion:**

Despite wide limits of agreement in Bland-Altman analysis, the SK-5500B showed good agreement and highly comparable repeatability with the Icare IC200 in both sitting and supine positions, supporting its potential as a practical clinical alternative.

## 1 Introduction

The accurate and reliable measurement of intraocular pressure (IOP) is a cornerstone of ophthalmic practice, particularly in the diagnosis and management of glaucoma, where IOP remains the primary modifiable risk factor (Lee et al., 2020). Goldmann applanation tonometry (GAT) is universally acknowledged as the gold standard for IOP assessment. However, its utility is constrained by the requirement for a seated position at a slit lamp, topical anesthesia, and a skilled operator (Whitacre and Stein, 1993). This limitation becomes critically relevant when evaluating IOP fluctuations influenced by physiological changes, such as those associated with body posture. Substantial evidence indicates that IOP is typically higher in a supine position compared to a seated position, a phenomenon with potential implications for understanding nocturnal IOP peaks and glaucoma progression (Galin et al., 1963; Linder et al., 1988). Consequently, there is a growing clinical need for tonometers that can provide accurate and reliable measurements in various body positions, beyond the conventional seated posture. Portable, hand-held tonometers, such as rebound tonometers, have emerged as practical alternatives for such scenarios (Muttuvelu et al., 2012; Dekking and Coster, 1967). Among these, the Icare IC200 (Icare Finland Oy, Vantaa, Finland) is a well-established device, with its performance characteristics and good agreement with GAT documented in the literature (Badakere et al., 2021; Zhang et al., 2025; Vieyres et al., 2025). Quantitative validation studies report a mean difference as low as 0.31 ± 1.43 mmHg and limits of agreement (LoA) within approximately ±3 mmHg when compared to GAT, alongside high concordance (Lin’s coefficient up to 0.95) and intraclass correlation coefficients (ICC > 0.9) (Vieyres et al., 2025; Rajagopal et al., 2025). Furthermore, it demonstrates clinically useful diagnostic accuracy, with reported sensitivity and specificity of 86% and 94.6%, respectively (Vieyres et al., 2025). In contrast, the SK-5500B (Shangbang, Inc., China) has been introduced as a newer rebound tonometer with potential to enhance accessibility and facilitate broader clinical deployment, necessitating further comparative evaluation against established devices. Before these tonometers can be confidently deployed for postural IOP studies or for assessing patients who cannot be positioned at a slit lamp, a rigorous evaluation of their measurement agreement and repeatability in both sitting and supine positions is imperative. Such a comparison is essential to determine whether these two tonometers are interchangeable in clinical and research settings and to validate their consistency across different postures.

Therefore, the purpose of this study is to evaluate and compare the intra-device repeatability and inter-device agreement of the SK-5500B and the Icare IC200 rebound tonometers for IOP measurement in both sitting and supine positions.

## 2 Materials and methods

### 2.1 Subjects

The study protocol received approval from the Institutional Review Board of the Joint Shantou International Eye Center of Shantou University and The Chinese University of Hong Kong (JSIEC) and adhered to the principles outlined in the Declaration of Helsinki. Prior to participation in any study-related procedures, written informed consent was obtained from all subjects after a comprehensive explanation of the study’s nature and potential risks.

Adult volunteers were recruited from the patient and staff population at the JSIEC. Eligible participants were required to be at least 18 years of age and had no severe spinal, neck, or systemic conditions that could impair their ability to comfortably and safely assume the required sitting and supine positions for IOP measurement. Exclusion criteria included the use of contact lenses within 12 h preceding the study measurements, any current ocular injury or trauma, active ocular infection, or a history of ocular infection within the past 3 months. These exclusions were implemented to ensure the integrity of the corneal surface and the accuracy of tonometric readings. All enrolled subjects underwent a single study visit, during which the IOP measurements were performed.

### 2.2 Tonometers

The IOP measurements in this study were performed using two handheld tonometers that both operate on the principle of rebound tonometry. This methodology involves a lightweight, magnetized probe against the cornea and measuring the characteristics of its deceleration upon impact (Cervino, 2006). The Icare IC200 (Icare Finland Oy, Vantaa, Finland) is a portable tonometer designed for clinical use. It is equipped with an adjustable forehead support to stabilize the device during measurement, an LCD display, and a control interface with four buttons that allow the operator to navigate the settings menu, initiate measurements, and review historical data. For each IOP reading, the device obtains six consecutive measurements. The built-in software then excludes statistical outliers and calculates a final IOP value based on the average of the remaining four measurements, with each individual reading and the final average displayed on the screen (Badakere et al., 2021).

The SK-5500B (Shangbang, Inc., China) is another rebound tonometer that functions on similar principle to the Icare tonometers. Similar to the Icare IC200, it is a self-contained, portable instrument intended for operator-assisted IOP assessment in a clinical setting. It performs a series of measurements and automatically processes the data to provide a final averaged IOP reading. A key feature of both the Icare IC200 and the SK-5500B is an integrated reliability indicator. This system provides immediate feedback on the quality of the measurement through a color-coded signal. A green light indicates a reliable measurement with low variability among the individual readings, while a yellow light signifies a measurement with significant variation, suggesting lower reliability. A red light is displayed when the device alignment is incorrect or the measurement distance to the eye is improper, prompting the operator to repeat the procedure (Sánchez Pavón et al., 2020).

### 2.3 Procedure

Following the provision of informed consent, all enrolled subjects underwent a comprehensive baseline ophthalmic examination. For each participant, data were first collected from both eyes, including an IOP measurement obtained with a non-contact tonometry (NCT) to serve as a reference value, as well as central corneal thickness (CCT) and axial length (AL) measurements acquired using the IOLMaster (De Bernardo et al., 2018). Based on the IOP reading from the NCT, subjects were subsequently categorized into one of four study groups: a low-IOP group (IOP < 10 mmHg), a normal-IOP group (IOP 10–21 mmHg), a moderately elevated IOP group (IOP 22–30 mmHg), and a high-IOP group (IOP > 30 mmHg) (Chen et al., 2019).

The IOP measurement protocol adhered to strict positioning standards. For seated measurements, subjects were instructed to maintain an upright posture for the upper body and head, with arms resting naturally and legs bent at a right angle. For supine measurements, subjects lay flat with their arms and legs relaxed and extended alongside the body, ensuring the head remained aligned with the body’s axis (Najmanová et al., 2020). A universal requirement for both positions dictated that subjects keep their eyes open, refraining from refractive correction, and fixating on a distant target directly ahead. All IOP measurements with the rebound tonometers were performed at a distance of 5–8 mm from the cornea, with the probe held perpendicularly to the central corneal surface for a straight-on measurement (Badakere et al., 2021).

The sequence of posture (sitting or supine), the order of application for the two rebound tonometers (Icare IC200 and SK-5500B), and the assignment of the examining operator were randomized for each participant. This randomization was performed using a computer-generated random number list prior to data collection in order to mitigate potential order effects and minimize operator-related bias in the IOP readings. Two different experienced ophthalmologists, who were masked to each other’s readings and the reference NCT values, performed the IOP measurements. For each device and in each body position, a total of six consecutive IOP readings were taken, from which the device-calculated average was recorded. A mandatory rest period of 5 min was enforced between measurements in different positions to allow for the stabilization of IOP and to mitigate the potential influence of the preceding measurement (Sang et al., 2024). This randomized, masked, and spaced protocol was designed to ensure the reliability and validity of the comparative data.

### 2.4 Statistical analysis

The sample size was calculated with reference to a previous study using an Icare tonometer (Chen et al., 2019). For a single-group design aiming to estimate the 95% confidence interval of a mean, with a margin of error (δ) set at 2.95 mmHg and a two-sided α level of 0.05, the calculation was performed using PASS 2023 (Power Analysis and Sample Size Software 2023. NCSS, LLC. Kaysville, Utah, United States). This calculation yielded a minimum required sample size of 59 subjects. We finally included 59 volunteers to guarantee an adequate sample size for statistical analysis.

SPSS27.0 (IBM, Chicago, United States) and Graph Pad Prism 10.1.2 (GraphPad Software, San Diego, CA, United States) software were used to process data. The measurement data such as IOP values were tested for normality. The continuous variables conforming to the normal distribution were described by the mean and standard deviation (SD). A descriptive statistical analysis was conducted to calculate the demographic characteristics of the study cohort. The data are presented as the mean, along with the standard deviation and the 95% confidence interval. A one-way analysis of variance (ANOVA) was used to compare the mean IOP measurements between the two IOP measurement methods. Pearson correlation and intraclass correlation coefficient (ICC) were employed to evaluate the correlation, agreement, and repeatability between the two tonometers. The Pearson correlation was used to assess the correlation between the measurements from different tonometers across all body positions. Furthermore, the same test was used to evaluate the correlation of CCT with measurements from both tonometers, as well as the correlation of AL with the measurements. A Pearson correlation coefficient of r = 0–0.2 was interpreted as a very weak or no correlation, r = 0.2–0.4 as a weak correlation, r = 0.4–0.6 as a moderate correlation, r = 0.6–0.8 as a strong correlation, and r = 0.8–1.0 as a very strong correlation. The ICC ranges from 0 to 1. Typically, an ICC < 0.2 indicates poor agreement, 0.2–0.4 indicates fair agreement, 0.4–0.6 indicates moderate agreement, 0.6–0.8 indicates strong agreement, and 0.8–1.0 indicates very strong agreement. Bland-Altman analysis was used to assess the agreement between the IOP values obtained by the two measurement methods (Bland and Altman, 1986; Fleiss et al., 1973). A *P*-value of less than 0.05 was considered statistically significant.

## 3 Results

### 3.1 Patient characteristics

Patient demographic and baseline characteristics are summarized in Table 1. The study included a total of 97 eyes from 59 subjects (31 males and 28 females) with a mean age of 50.8 ± 18.2 years (range: 23–84 years). Among these patients, 8 eyes with an IOP < 10 mmHg, 50 eyes with an IOP within 10–21 mmHg, 19 eyes within 22–30 mmHg, and 20 eyes >30 mmHg.

**TABLE 1:** Baseline demographics and mean IOP of the study population.

Parameters	N
No. patients (M/F)	59 (31/28)
No. eyes (R/L)	97 (50/47)
Mean age, mean ± SD (range), y	50.8 ± 18.2 (23–84)
CCT, mean ± SD (range), μm	542.9 ± 38.3 (412–660)
AL, mean ± SD (range), mm	23.9 ± 1.5 (21.6–28.8)
NCT, mean ± SD (range), mmHg	22.3 ± 12.9 (6.0–77.0)
SK-5500B, mean ± SD (range), mmHg	24.1 ± 12.5 (4.6–70.0)
Icare IC200, mean ± SD (range), mmHg	24.0 ± 15.1 (3.4–78.7)

SD, standard deviation; CCT, central corneal thickness; AL, axial length.

### 3.2 Comparison between SK-5500B and Icare IC200 IOP measurements

**FIGURE 1:**
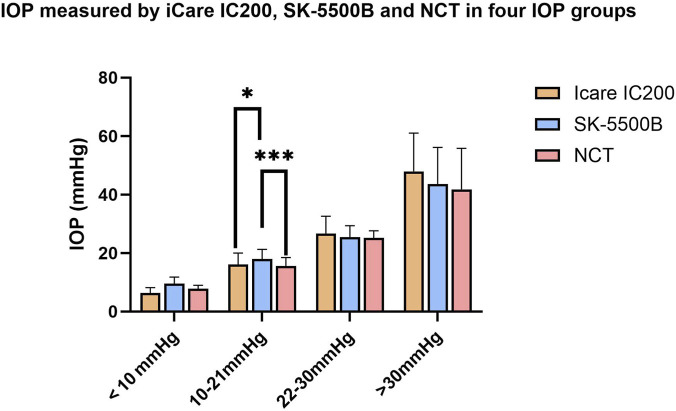
IOP measured by Icare IC200, SK-5500B and NCT in four IOP groups.

The mean IOP measured by NCT, SK-5500B and Icare IC200 were 22.3 ± 12.9 mmHg (range: 6.0–77.0 mmHg), 24.1 ± 12.5 mmHg (range: 4.6–70.0 mmHg) and 24.0 ± 15.1 mmHg (range: 3.4–78.7 mmHg), respectively (Table 1). No significant difference was found among the IOP measured by three methods in IOP < 10 mmHg, IOP 10–21 mmHg, IOP 22–30 mmHg and IOP > 30 mmHg groups (*P* > 0.05, one-way ANOVA). Post-hoc analyses with Bonferroni correction were subsequently performed to identify specific pairwise differences within each IOP group. Significant inter-device differences were identified in the normal-IOP groups. In the IOP 10–21 mmHg group, significant differences were observed both between the SK-5500B and NCT (*P* = 0.001) and between the SK-5500B and the Icare IC200 (*P* = 0.017). No statistically significant pairwise differences were found among the three devices in the IOP < 10 mmHg, IOP 22–30 mmHg and IOP > 30 mmHg groups (all corrected *P* > 0.05). These results are summarized in Figure 1.

Table 2 summarizes the IOP measurements obtained by the two instruments in the different positions, showing that there were statistically significant differences between the IOP measurements obtained by the same device (all *P* < 0.01).

**TABLE 2:** Comparison between Icare IC200 and SK-5500B IOP measurements.

Statistic	Operator 1		Operator 2	
	SK-5500B	Icare IC200	SK-5500B	Icare IC200
Sitting				
Mean ± SD	22.4 ± 12.2	21.9 ± 14.5	22.8 ± 12.6	21.7 ± 14.4
95% CI	19.9–24.9	19.0–24.8	20.2–25.3	18.8–24.6
Range	5.0–67.0	3.6–77.3	4.0–70.0	3.7–77.4
Supine				
Mean ± SD	26.0 ± 12.7	26.2 ± 15.7	25.2 ± 13.4	26.2 ± 16.1
95% CI	23.4–28.5	23.0–29.3	22.5–27.9	23.0–29.4
Range	4.6–67.5	3.5–78.7	6.9–68.4	3.4–77.7
*P* value#	<0.01	<0.01	<0.01	<0.01

CI, confidence interval. *P* value#, differences between the IOP, measurements obtained by the same device in different position (paired t-test).

**FIGURE 2:**
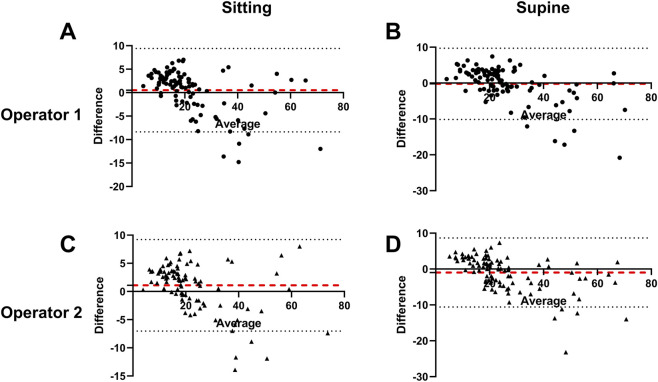
Bland–Altman analysis of agreement in IOP measurements between the Icare IC200 and SKB-5500B tonometers. The red dashed line represents the mean difference, and the black dashed lines represent the limits of agreement (±1.96 SD). **(A)** Operator 1, sitting position. **(B)** Operator 1, supine position. **(C)** Operator 2, sitting position. **(D)** Operator 2, supine position. All values are in mmHg.

The Bland-Altman plot in Figure 2 depicts the paired differences to the average IOP between the Icare IC200 and SKB-5500B. The analysis revealed a consistent pattern of agreement across different operators and positions. For Operator 1, the mean difference (bias, IOP_SK-5500B–IOP_Icare IC200) was 0.52 ± 4.54 mmHg with 95% limits of agreement (LoA) ranging from −8.37 mmHg to 9.41 mmHg in the sitting position, and −0.21 ± 5.06 mmHg (LoA: −10.13 to 9.71 mmHg) in the supine position. Similarly, for Operator 2, the bias was 1.10 ± 4.15 mmHg (LoA: −7.04–9.22 mmHg) in the sitting position and −0.97 ± 4.90 mmHg (LoA: −10.57 to 8.64 mmHg) in the supine position. The agreement between the two tonometers was clinically acceptable, as the proportion of SK-5500B measurements within ±3 mmHg of the Icare IC200 reference exceeded half, with specific rates of: 54.6% (Operator 1, sitting), 56.7% (Operator 1, supine), 49.5% (Operator 2, sitting), and 51.6% (Operator 2, supine).

**FIGURE 3:**
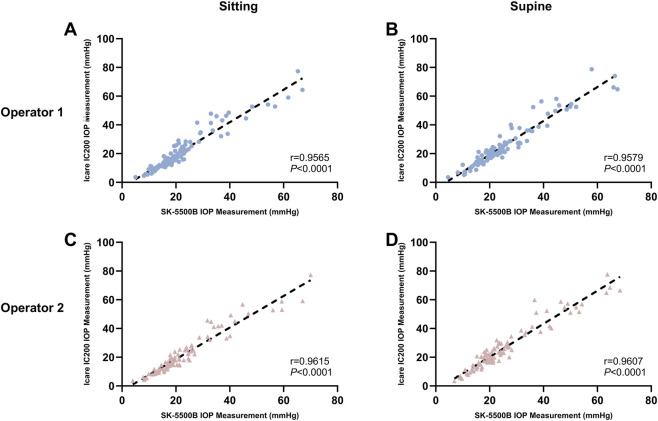
Correlation of IOP measurements between the Icare IC200 and the SKB-5500B tonometers. **(A)** Sitting position, Operator 1. **(B)** Supine position, Operator 1. **(C)** Sitting position, Operator 2. **(D)** Supine position, Operator 2. All values are in mmHg.

The paired IOP measurements between the SK-5500B and Icare IC200 showed strong and significant correlations (all *P* < 0.0001) across all conditions (Figure 3). The Pearson correlation coefficients were r = 0.9565 for Operator 1 (sitting), r = 0.9579 for Operator 1 (supine), r = 0.9615 for Operator 2 (sitting), and r = 0.9607 for Operator 2 (supine).

**FIGURE 4:**
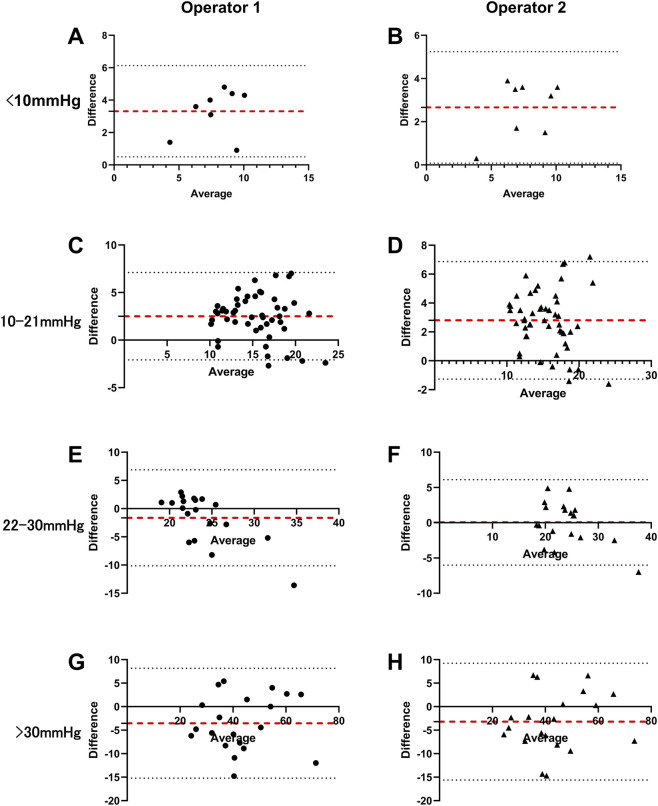
Agreement between the two IOP measurements in the sitting position across four IOP groups. The red dashed line represents the mean difference, and the black dashed lines represent the limits of agreement (±1.96 SD). The diagrams are organized in a 4 × 2 layout: rows represent different IOP groups (from top to bottom: low, normal, moderately elevated, and higher), and columns represent different operators (Operator 1 and Operator 2). **(A,C,E,G)** Bland-Altman plots for Operator 1. **(B,D,F,H)** Bland-Altman plots for Operator 2.

**FIGURE 5:**
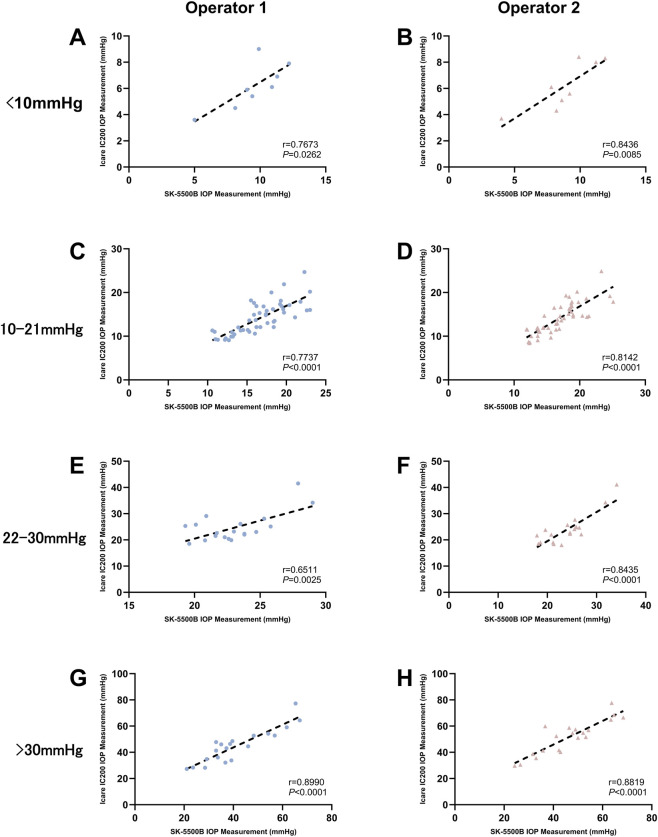
Correlation between SK-5500B and Icare IC200 measurement in four IOP groups (sitting). Rows represent different IOP groups (from top to bottom: low, normal, moderately elevated, and higher), and columns represent different operators [Operator 1 **(A, C, E, G)** and Operator 2 **(B, D, F, H)**].

**FIGURE 6:**
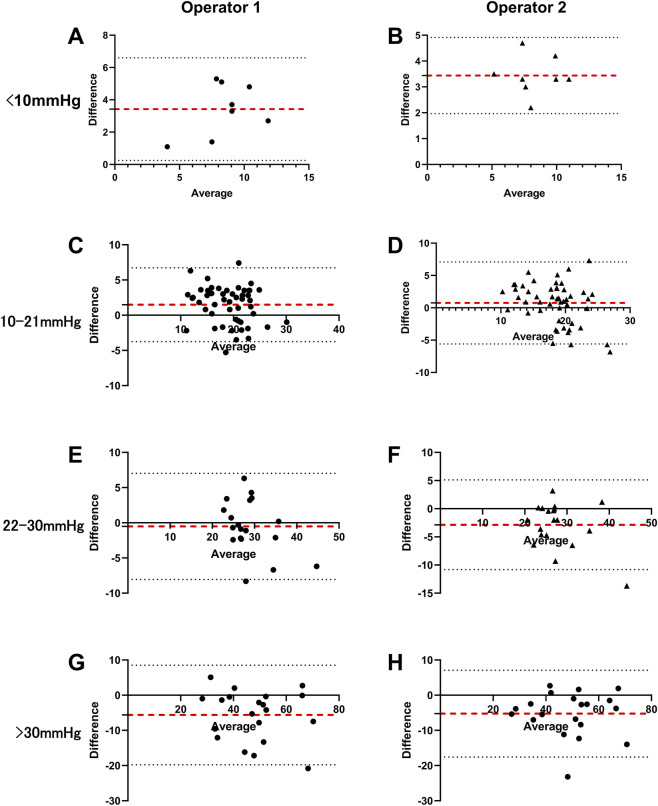
Agreement between the two IOP measurements in the supine position across four IOP groups. The red dashed line represents the mean difference, and the black dashed lines represent the limits of agreement (±1.96 SD). The diagrams are organized in a 4 × 2 layout: rows represent different IOP groups (from top to bottom: low, normal, moderately elevated, and higher), and columns represent different operators (Operator 1 and Operator 2). **(A,C,E,G)** Bland-Altman plots for Operator 1. **(B,D,F,H)** Bland-Altman plots for Operator 2.

**FIGURE 7:**
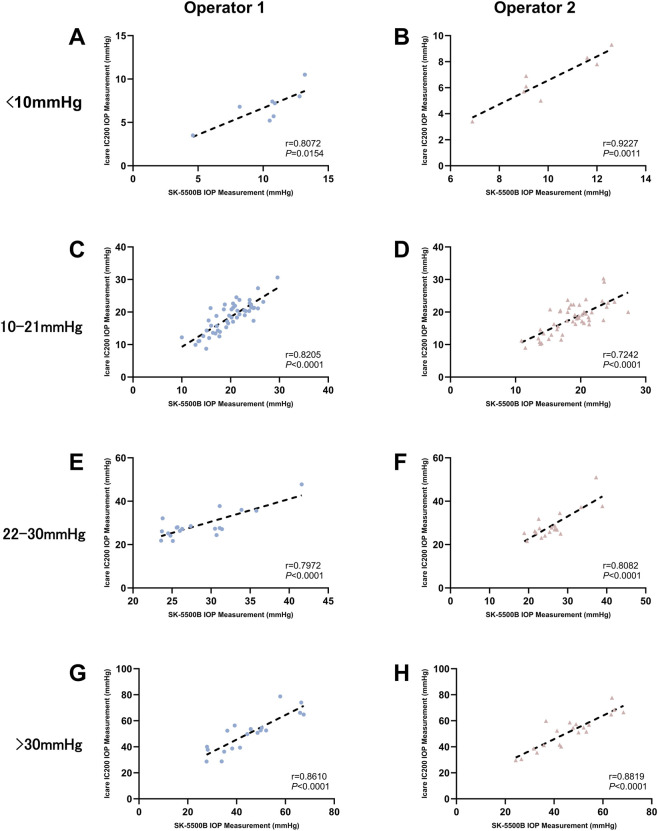
Correlation between SK-5500B and Icare IC200 measurement in four IOP groups (supine). Rows represent different IOP groups (from top to bottom: low, normal, moderately elevated, and higher), and columns represent different operators [Operator 1 **(A, C, E, G)** and Operator 2 **(B, D, F, H)**].

The agreement and correlation between SK-5500B and Icare IC200 measurements were illustrated in Figures 4–7 in four IOP groups and different posture, respectively. Bland-Altman analysis revealed that the SK-5500B tended to overestimate IOP compared to the Icare IC200 in the low IOP group, while it tended to underestimate IOP in the high IOP group. Furthermore, Pearson correlation analysis demonstrated strong and statistically significant correlations between the two tonometers across all IOP groups (all r > 0.8, *P* < 0.05). Bias and LoA are provided in Table 3.

**TABLE 3:** Summary of the Bland-Altman plots.

Condition	Bias	Limits of agreement		With ±3 mmHg#	>±5 mmHg##
		Lower	Upper		
IOP < 10 mmHg					
Sitting, operator 1	3.31 ± 1.44	0.50	6.13	25%	0%
Sitting, operator 2	2.66 ± 1.32	0.08	5.24	37.5%	0%
Supine, operator 1	3.43 ± 1.62	0.25	6.60	37.5%	25%
Supine, operator 2	3.43 ± 0.75	1.96	4.91	25%	0%
IOP 10–21 mmHg					
Sitting, operator 1	2.51 ± 2.34	−2.08	7.11	52.4%	16.7%
Sitting, operator 2	2.80 ± 2.08	−1.27	6.87	50%	16.7%
Supine, operator 1	1.49 ± 2.67	−3.75	6.72	50%	7.1%
Supine, operator 2	0.75 ± 3.24	−5.59	7.10	54.8%	9.5%
IOP 22–30 mmHg					
Sitting, operator 1	−1.63 ± 4.34	−10.13	6.88	73.7%	26.3%
Sitting, operator 2	0.05 ± 3.09	−6.01	6.11	73.7%	5.3%
Supine, operator 1	−0.52 ± 3.85	−8.05	7.02	57.9%	21.1%
Supine, operator 2	−2.86 ± 4.07	−10.83	5.11	52.6%	21.1%
IOP > 30 mmHg					
Sitting, operator 1	−3.53 ± 5.97	−15.22	8.16	30%	50%
Sitting, operator 2	−3.20 ± 6.34	−15.63	9.24	30%	60%
Supine, operator 1	−5.62 ± 7.22	−19.77	8.53	45%	50%
Supine, operator 2	−5.25 ± 6.30	−17.58	7.10	45%	45%

Bias, calculated as the IOP, difference (SK-5500B minus Icare IC200). With ±3 mmHg#, clinical acceptable differences. >±5 mmHg##, a significant variability.

### 3.3 Repeatability of Icare IC200 and SKB-5500B

A total of 32 eyes from 24 healthy subjects (12 men, 12 women) who were 52.8 ± 16.9 years old (range 24–76 years) were included in repeatability analysis.

Each IOP measurement comprised three independent readings taken with both the SK-5500B and the Icare IC200. Repeated-measures ANOVA was applied to assess the repeatability of measurements between the different operators. The resulting *P*-values were all >0.05, indicating no statistically significant difference in repeatability of SK-5500B and Icare IC200.

The CV for the two tonometers was less than 10%, with a Sw close to 1 mmHg. The ICC showed values greater than 0.99 for all tonometers, thus showing high agreement (Table 4).

**TABLE 4:** Intra-operator repeatability for IOP measured in different tonometers and postures.

Device	Mean ± SD	Range	Sw	Prec	Rep	CV	ICC
Sitting							
SK-5500B	23.6 ± 15.3	7.6–69.2	0.88	1.73	2.44	2.46%	0.995
Icare IC200	20.9 ± 16.3	3.8–65.8	0.86	1.69	2.38	4.12%	0.997
Supine							
SK-5500B	27.1 ± 16.4	8.2–69.8	1.15	2.25	3.19	4.24%	0.993
Icare IC200	25.4 ± 18.7	4.8–74.0	0.96	1.88	2.66	3.78%	0.995
P value#	0.754						
P value##	0.299						
P value###	0.692						
P value####	0.088						

Sw, within-subject standard deviation; Prec, precision; Rep, repeatability; CV, co-efficient of variation; ICC, intraclass correlation coefficient (two-way mixed effects model for absolute agreement, single measures). P value#, difference among three measurements with SK-5500B in sitting position (Repeated-measures ANOVA). P value##, difference among three measurements with SK-5500B in supine position (Repeated-measures ANOVA). P value###, difference among three measurements with Icare IC200 in sitting position (Repeated-measures ANOVA). P value####, difference among three measurements with Icare IC200 in supine position (Repeated-measures ANOVA).

The means of paired IOP measurement for all two operators were compared, using paired t-test. The observed difference in inter-operator variability was not statistically significant (all *P* > 0.05), indicating no significant inter-operator variability in the measurement of IOP with the SK-5500B and Icare IC200. The CV for the two tonometers was less than 10%, with a Sw close to 1 mmHg. The ICC showed values greater than 0.99 for all tonometers, thus showing high agreement. These results are summarized in Table 5.

**TABLE 5:** Inter-operator repeatability for IOP measured with Icare IC200 and SK-5500B.

Device	Mean ± SD	Range	Sw	Prec	Rep	CV	ICC
Sitting							
SK-5500B	25.5 ± 16.0	8.0–69.2	1.10	2.16	3.05	4.31%	0.993
Icare IC200	23.3 ± 17.8	3.8–74.0	0.89	1.74	2.47	3.82%	0.996
Supine							
SK-5500B	25.2 ± 16.03	7.6–69.8	0.93	1.82	2.58	3.69%	0.995
Icare IC200	23.0 ± 17.6	4.3–72.1	0.93	1.82	2.58	4.04%	0.996
P value#	0.283						
P value##	0.062						

P value#, difference between operator1 and operator2 with SK-5500B (Paired t-test). P value##, difference between operator1 and operator2 with Icare IC200 (Paired t-test).

### 3.4 Correlation between Icare IC200 and SKB-5500B, correlation between CCT, AL and IOP measurements

There was a strong positive correlation between the measurements obtained by the Icare IC200 and SKB-5500B in all positions (all r ≥ 0.699, all *P* < 0.05). The CCT ranged from 412 to 660 µm (mean: 542.9 ± 38.3 µm) and the AL ranged from 21.6 to 28.8 mm (mean: 23.9 ± 1.5 mm), as summarized in Table 1. Multivariate ANOVA demonstrated that IOP measurements from both the SK-5500B and Icare IC200 were not significantly influenced by variations in CCT (*P* = 0.178, Wilks lambda multivariate statistic) or AL (*P* = 0.439, Wilks lambda multivariate statistic).

The correlations of CCT and AL with the inter-device IOP difference (Icare IC200 vs. SK-5500B) across all positions are summarized in Table 6. There was a very weak, not statistically significant, correlation between the CCT and the difference between the two tonometers in all positions (all *P* > 0.05). The correlations between the AL and the difference between the Icare IC200 and SKB-5500B in all positions are shown in Table 6. In the same way, there was very weak correlation between the AL and the difference between the two tonometers in all positions (all *P* > 0.05).

**TABLE 6:** Person’s correlation coefficient (r) between difference in IOP measurements obtained in different positions between Icare IC200 and SK-5500B, and central corneal thickness (CCT) or axial length (AL).

Difference between Icare IC200 and SK-5500B		
Parameter	Sitting	Supine
Operator 1		
CCT	0.14	0.14
AL	−0.05	0.02
Operator 2		
CCT	0.05	0.04
AL	0.02	−0.09

## 4 Discussion

The accurate measurement of IOP is important in managing many ophthalmic conditions. GAT is universally regarded as the gold standard for IOP assessment (Mohan et al., 2014). Technological advancements have facilitated the development of digital GAT, such as the Huvitz HT5000, which retains fundamental mechanical principles while incorporating modern features like digital readouts (Kratz et al., 2025). Despite these improvements, a fundamental limitation of this technique remains its requirement for the patient to be in a seated position. This poses a significant challenge in clinical scenarios where a slit lamp is unavailable or where the patient cannot be positioned upright, such as during trauma assessments in the emergency department. Under these circumstances, portable handheld tonometers become indispensable.

The Icare IC200, as the newest generation of rebound tonometer, is the product of a developmental series that began with the initial prototype described by Kontiola in 1997 (Nakakura, 2018; Kontiola, 1996). Since its introduction, the reliability and clinical utility of the Icare IC200 rebound tonometer have been substantiated across multiple patient populations and clinical contexts. For example, Vieyres et al. (2025) demonstrated that the Icare IC200 provides consistent IOP measurements in adults, independent of CCT or body mass index. They highlighted its portability and ease of use, endorsing its suitability for routine ophthalmic practice. Further supporting its versatility, Rajagopal et al. (2025) reported good agreement among GAT, Icare IC200, and NCT in post-vitreoretinal surgery patients. Importantly, Icare IC200 was successfully used in all eyes during the immediate postoperative period, irrespective of corneal status, whereas both GAT and NCT were unrecordable in a subset of patients due to corneal alterations. While a study in a pediatric population found that the Icare IC200 tended to overestimate IOP compared to Perkins tonometry, the two devices still demonstrated a moderate level of agreement (Stoddard-Bennett et al., 2022).

In parallel, the SK-5500B (Shangbang, Inc., China) has emerged as a new, Chinese produced rebound tonometer. A key feature of this portable device is its ability to measure IOP in both sitting and supine positions, facilitated by a probe head with 200 degrees of positional freedom, enhancing its adaptability in challenging clinical scenarios. However, robust evidence regarding its agreement with established tonometers and its overall clinical performance remains scarce. To our knowledge, a comprehensive evaluation of the agreement and repeatability of the SK-5500B is lacking, and the present study aims to fill this knowledge gap by providing the first comprehensive evaluation of the agreement and repeatability of the SK-5500B compared to the Icare IC200.

The primary finding reveals the strong correlation between the SK-5500B and the well-established Icare IC200. The Pearson correlation coefficients were consistently high (>0.95) across all conditions (both positions and both operators), indicating that the two tonometers exhibit an excellent synchronized response to changes in IOP. However, the Bland-Altman analysis provides a more nuanced perspective on their clinical interchangeability. Despite the strong correlation, the 95% limits of agreement were wide, and approximately half of the measurement differences fell within the clinically acceptable margin of ±3 mmHg in the normal IOP range. A more detailed analysis stratified by IOP groups revealed that, the SK-5500B overestimating IOP in the low IOP group (<10 mmHg) while underestimating it in the high IOP group (>30 mmHg). Notably, in the high IOP group, nearly half of the differences exceeded ±5 mmHg, which indicates substantial variability. Nonetheless, it should be noted that the relatively small sample sizes in these extreme IOP subgroups may limit the robustness of these observations.

Consistent with existing literature (Schweier et al., 2013; Tarkkanen and Leikola, 1967; Jain and Marmion, 1976), this study confirmed a statistically significant increase in IOP when subjects moved from a sitting to a supine position. The ability of both the SK-5500B and Icare IC200 to reliably detect this postural IOP fluctuation underscores their value in research on nocturnal IOP elevation and in the clinical assessment of patients who cannot maintain an upright position for standard tonometry.

Furthermore, both tonometers demonstrated excellent repeatability. The high ICC values (≥0.99) for both intra-operator and inter-operator assessments indicate that the measurements are highly consistent, regardless of who is performing the test. This high level of repeatability is essential for a clinical instrument, as it ensures that measured changes in IOP reflect true physiological variations rather than measurement error.

An important finding was the lack of a significant correlation between the inter-device measurement difference and key ocular biometric parameters like CCT and AL. This suggests that the agreement between the SK-5500B and Icare IC200 is robust and not significantly confounded by variations in corneal thickness or AL.

This study has several limitations that must be acknowledged. First, the agreement of the SK-5500B was evaluated against the Icare IC200 rather than the gold standard, GAT. However, it is important to note that the Icare IC200 itself has been previously validated and demonstrates good agreement with GAT in studies (Rajagopal et al., 2025), which indirectly supports the clinical relevance of our findings. Second, participants were not systematically evaluated for glaucoma with a comprehensive diagnostic examination independent of their IOP values. While this was consistent with our primary aim of comparing tonometric agreement, the potential influence of undiagnosed glaucoma on the measurements cannot be ruled out. Third, our study cohort was restricted to adult participants (≥18 years of age). Consequently, the performance of the SK-5500B in pediatric populations, who often present greater challenges in obtaining cooperative IOP measurements, remain unknown and warrant future investigation. Fourth, we did not systematically assess or control for potential confounders such as lens status and anterior chamber depth (ACD), both of which may influence IOP measurements. For example, Grewal et al. (2016) reported that the ICCs between the Icare tonometer and both GAT and Tonopen tonometers were lower in aphakic eyes (ranging from 0.68 to 0.79) compared to those in pseudophakic eyes (0.85–0.91) and phakic eyes (0.85–0.94). The study by Ye et al. (2022) reported a weak positive correlation between ACD and IOP as measured by the Icare tonometer, with a correlation coefficient of 0.291 (*P* = 0.024). Since these factors could affect tonometric readings, their absence from our analysis means we cannot exclude their potential confounding effect on the observed agreement between the two devices. Whether and how these factors consistently influence IOP measurement warrants further investigation.

While the results are promising, future studies are warranted. Investigations involving more diverse populations, such as those with corneal pathologies, post-refractive surgery eyes, or different age groups, would help to further validate the utility of the SK-5500B tonometer across a broader spectrum of clinical scenarios.

Finally, our study assessed agreement at a single time point, thus the dynamic performance of the SK-5500B in tracking IOP fluctuations over time remains unverified. Future longitudinal studies, such as those evaluating diurnal or treatment-induced IOP variations, are needed to confirm if the observed agreement remains consistent during dynamic IOP changes, thereby further validating its utility for long-term monitoring.

## 5 Conclusion

In conclusion, our study provides the first comprehensive evaluation of the SK-5500B rebound tonometer against the established Icare IC200. Despite wide limits of agreement in Bland-Altman analysis, the SK-5500B tonometer demonstrated good agreement with the Icare IC200 across a wide IOP range, supporting its potential as a clinically effective alternative. The measurements provided by the SK-5500B were highly repeatable and closely aligned with those from the Icare IC200. Furthermore, the differences between the two tonometers showed no significant correlation with CCT or AL. Future studies involving diverse patient populations, including different age groups and those with ocular conditions, are warranted to further validate the utility of these rebound tonometers in long-term patient management.

## Data Availability

The original contributions presented in the study are included in the article/supplementary material, further inquiries can be directed to the corresponding authors.
